# (*E*)-2-Methyl-6-[(1-phenyl-1*H*-pyrazol-4-yl)methyl­idene]cyclo­hexa­none

**DOI:** 10.1107/S160053681102126X

**Published:** 2011-06-11

**Authors:** Abdullah M. Asiri, Hassan M. Faidallah, Seik Weng Ng

**Affiliations:** a Chemistry Department, Faculty of Science, King Abdul Aziz University, Jeddah 21589, Saudi Arabia; b Department of Chemistry, University of Malaya, 50603 Kuala Lumpur, Malaysia

## Abstract

The asymmetric unit of the title compound, C_17_H_18_N_2_O, contains two independent mol­ecules. In both, the cyclo­hexane ring adopts a flattened chair conformation, and the 3- and 4-methyl­ene C atoms as well as the methyl C atoms are disordered over two positions, the occupancy of the major component being 68 (1)% in one mol­ecule and 64 (1)% in the other. The phenyl and pyrazole rings in both mol­ecules are approximately coplanar, the r.m.s. deviations being 0.048 and 0.015 Å, respectively. Weak inter­molecular C—H⋯O hydrogen bonding is present in the crystal structure.

## Related literature

For a recent report on similar heterocylic compounds derived from substituted 1-phenyl­pyrazole-4-carboxaldehydes>, see: Asiri & Khan (2010 ▶).
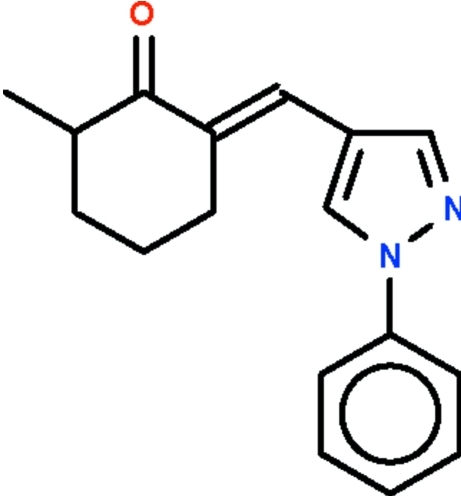


## Experimental

### 

#### Crystal data

C_17_H_18_N_2_O
*M* *_r_* = 266.33
Triclinic, 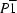

*a* = 6.1152 (8) Å
*b* = 10.3757 (13) Å
*c* = 22.734 (3) Å
α = 77.542 (2)°
β = 89.667 (2)°
γ = 78.510 (2)°
*V* = 1379.2 (3) Å^3^
*Z* = 4
Mo *K*α radiation
μ = 0.08 mm^−1^
*T* = 100 K
0.20 × 0.20 × 0.05 mm

#### Data collection

Bruker SMART APEX diffractometer
14492 measured reflections
4890 independent reflections
3235 reflections with *I* > 2σ(*I*)
*R*_int_ = 0.051

#### Refinement

*R*[*F* ^2^ > 2σ(*F* ^2^)] = 0.062
*wR*(*F* ^2^) = 0.172
*S* = 1.03
4890 reflections
408 parameters
47 restraints
H-atom parameters constrained
Δρ_max_ = 0.40 e Å^−3^
Δρ_min_ = −0.27 e Å^−3^

### 

Data collection: *APEX2* (Bruker, 2009 ▶); cell refinement: *SAINT* (Bruker, 2009 ▶); data reduction: *SAINT*; program(s) used to solve structure: *SHELXS97* (Sheldrick, 2008 ▶); program(s) used to refine structure: *SHELXL97* (Sheldrick, 2008 ▶); molecular graphics: *X-SEED* (Barbour, 2001 ▶); software used to prepare material for publication: *publCIF* (Westrip, 2010 ▶).

## Supplementary Material

Crystal structure: contains datablock(s) global, I. DOI: 10.1107/S160053681102126X/xu5225sup1.cif

Structure factors: contains datablock(s) I. DOI: 10.1107/S160053681102126X/xu5225Isup2.hkl

Supplementary material file. DOI: 10.1107/S160053681102126X/xu5225Isup3.cml

Additional supplementary materials:  crystallographic information; 3D view; checkCIF report

## Figures and Tables

**Table 1:** Hydrogen-bond geometry (Å, °)

*D*—H⋯*A*	*D*—H	H⋯*A*	*D*⋯*A*	*D*—H⋯*A*
C9—H9⋯O1^i^	0.95	2.29	3.157 (4)	152
C26—H26⋯O2^ii^	0.95	2.30	3.224 (4)	164
C30—H30⋯O2^ii^	0.95	2.57	3.506 (4)	167

Symmetry codes: (i) 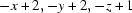; (ii) 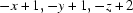.

**Fig. 1.**
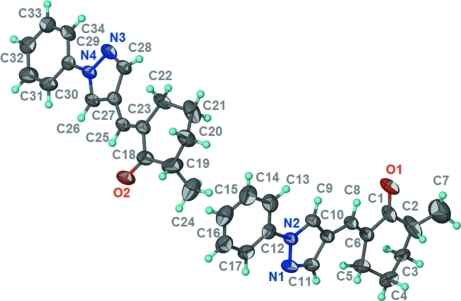
Thermal ellipsoid plot (Barbour, 2001) of the two independent molecules of C17H18N2O at the 70% probability level; hydrogen atoms are drawn as spheres of arbitrary radius. The minor disorder component is not shown.

## supplementary crystallographic information

### Comment

The hydrogen atoms of the α-methylene carbons of cyclohexanone are relatively acidic, and the compound can undergo an aldol-type condensation with an aldehyde. This study employs the aldehyde, 1-phenylpyrazole-4-carboxaldehyde, to react with 2-methylcyclohexanone to yield a new heterocyclic compound that is expected to possess useful biological actvity. The present structural study extends a recent report on similar heterocylic compounds derived from substituted 1-phenylpyrazole-4-carboxaldehydes (Asiri & Khan, 2010). In the two independent molecules of C_17_H_18_N_2_O (Scheme I), the cyclohexane ring adopts a flattened chair conformation; the phenylpyrazolyl substituent of the ring in both molecules, which is planar (r.m.s. deviation 0.048, 0.015 Å), is approximately co-planar with the mean plane passing through the cyclohexane ring (Fig.1). The methylene carbon that provided the acidic H atoms is *sp*^2^-hybridized in the product; the strain in the ring is now reflected in an ethylene fragment as well as in the methyl substituent adopting two orientations. The 3- and 4-methylene as well as the methyl C atoms are disordered, with the occupancy of the major component being 68 (1)% in one molecule and 64 (1)% in the other.

### Experimental

1-Phenylpyrazole-4-carboxaldehyde (1.72 g, 0.01 mol) in ethanol (20 ml) was added to 2-methylcyclohexanone (1.12 g, 0.01 mol) in 20% alcoholic potassium hydroxide (20 ml). The mixture was stirred for 6 h. The solid product that separated was collected and recrystallized from ethanol.

### Refinement

Carbon-bound H-atoms were placed in calculated positions (C–H 0.95 to 1.00 Å) and were included in the refinement in the riding model approximation, with *U*(H) set to 1.2 to 1.5*U*_eq_(C).

The 3- and 4-methylene as well as the methyl substituent of the cyclohexane ring is disordered over two positions; for one molecule, the occupancy of the major component refined to 68 (1)% and for the other, this refined to 64 (1)%. The carbon-carbon single-bond distances were restrained to 1.54–0.01 Å; for the methyl C atom, the 1,3-related distances were restrained to 2.51±0.01 Å. Additionally, the anisotropic temperature factors of the minor-component atoms were restrained to be nearly isotropic.

### Crystal data

**Table:**

C_17_H_18_N_2_O	*Z* = 4
*M**_r_* = 266.33	*F*(000) = 568
Triclinic, *P*1	*D*_x_ = 1.283 Mg m^−^^3^
Hall symbol: -P 1	Mo *K*α radiation, λ = 0.71073 Å
*a* = 6.1152 (8) Å	Cell parameters from 2004 reflections
*b* = 10.3757 (13) Å	θ = 3.5–23.0°
*c* = 22.734 (3) Å	µ = 0.08 mm^−^^1^
α = 77.542 (2)°	*T* = 100 K
β = 89.667 (2)°	Block, colorless
γ = 78.510 (2)°	0.20 × 0.20 × 0.05 mm
*V* = 1379.2 (3) Å^3^	

### Data collection

**Table:**

Bruker SMART APEX diffractometer	3235 reflections with *I* > 2σ(*I*)
Radiation source: fine-focus sealed tube	*R*_int_ = 0.051
graphite	θ_max_ = 25.1°, θ_min_ = 0.9°
ω scans	*h* = −7→7
14492 measured reflections	*k* = −12→12
4890 independent reflections	*l* = −27→27

### Refinement

**Table:**

Refinement on *F*^2^	Secondary atom site location: difference Fourier map
Least-squares matrix: full	Hydrogen site location: inferred from neighbouring sites
*R*[*F*^2^ > 2σ(*F*^2^)] = 0.062	H-atom parameters constrained
*wR*(*F*^2^) = 0.172	*w* = 1/[σ^2^(*F*_o_^2^) + (0.0685*P*)^2^ + 1.0076*P*] where *P* = (*F*_o_^2^ + 2*F*_c_^2^)/3
*S* = 1.03	(Δ/σ)_max_ < 0.001
4890 reflections	Δρ_max_ = 0.40 e Å^−^^3^
408 parameters	Δρ_min_ = −0.27 e Å^−^^3^
47 restraints	Extinction correction: *SHELXL97* (Sheldrick, 2008), Fc^*^=kFc[1+0.001xFc^2^λ^3^/sin(2θ)]^-1/4^
Primary atom site location: structure-invariant direct methods	Extinction coefficient: 0.0074 (17)

### Fractional atomic coordinates and isotropic or equivalent isotropic displacement parameters (Å^2^)

**Table:**

	*x*	*y*	*z*	*U*_iso_*/*U*_eq_	Occ. (<1)
O1	0.7546 (4)	1.1287 (2)	0.43105 (11)	0.0512 (7)	
O2	0.7707 (4)	0.4267 (2)	0.91788 (10)	0.0458 (6)	
N1	0.7423 (4)	0.5112 (2)	0.55321 (12)	0.0347 (6)	
N2	0.9381 (4)	0.5341 (2)	0.57342 (10)	0.0286 (6)	
N3	0.7798 (4)	0.9225 (3)	1.04505 (12)	0.0384 (7)	
N4	0.5931 (4)	0.8732 (2)	1.06408 (11)	0.0332 (6)	
C1	0.6219 (5)	1.0760 (3)	0.40999 (14)	0.0405 (8)	
C2	0.4788 (6)	1.1553 (4)	0.35595 (19)	0.0722 (14)	
H1	0.5484	1.0941	0.3295	0.087*	0.684 (10)
H1'	0.3958	1.2398	0.3667	0.087*	0.316 (10)
C5	0.3955 (5)	0.8869 (3)	0.41931 (14)	0.0389 (8)	
H5A	0.2891	0.8917	0.4522	0.047*	0.684 (10)
H5B	0.4408	0.7910	0.4171	0.047*	0.684 (10)
H5C	0.4396	0.8219	0.3931	0.047*	0.316 (10)
H5D	0.3372	0.8386	0.4566	0.047*	0.316 (10)
C6	0.6001 (5)	0.9351 (3)	0.43570 (14)	0.0330 (7)	
C8	0.7639 (5)	0.8587 (3)	0.47404 (13)	0.0322 (7)	
H8	0.8830	0.9015	0.4800	0.039*	
C9	0.9686 (5)	0.6585 (3)	0.54691 (13)	0.0304 (7)	
H9	1.0927	0.6963	0.5540	0.036*	
C10	0.7884 (5)	0.7216 (3)	0.50772 (13)	0.0297 (7)	
C11	0.6536 (5)	0.6233 (3)	0.51371 (13)	0.0331 (7)	
H11	0.5159	0.6363	0.4920	0.040*	
C12	1.0725 (5)	0.4378 (3)	0.61952 (13)	0.0309 (7)	
C13	1.2695 (5)	0.4635 (4)	0.64024 (15)	0.0450 (9)	
H13	1.3176	0.5441	0.6222	0.054*	
C14	1.3950 (6)	0.3724 (4)	0.68696 (16)	0.0514 (9)	
H14	1.5289	0.3908	0.7011	0.062*	
C15	1.3274 (6)	0.2545 (4)	0.71336 (16)	0.0493 (9)	
H15	1.4127	0.1923	0.7460	0.059*	
C16	1.1338 (6)	0.2282 (3)	0.69159 (15)	0.0472 (9)	
H16	1.0883	0.1464	0.7090	0.057*	
C17	1.0047 (5)	0.3195 (3)	0.64472 (14)	0.0381 (8)	
H17	0.8717	0.3007	0.6302	0.046*	
C18	0.8985 (5)	0.5011 (3)	0.89857 (14)	0.0402 (8)	
C19	1.0294 (6)	0.4779 (5)	0.84412 (18)	0.0658 (12)	
H19	1.0803	0.3777	0.8544	0.079*	0.636 (10)
H19'	1.1488	0.3974	0.8610	0.079*	0.364 (10)
C22	1.1215 (5)	0.6813 (3)	0.91155 (15)	0.0417 (8)	
H22A	1.2378	0.6422	0.9441	0.050*	
H22B	1.0741	0.7785	0.9110	0.050*	
C23	0.9246 (5)	0.6151 (3)	0.92600 (14)	0.0344 (7)	
C25	0.7668 (5)	0.6508 (3)	0.96382 (13)	0.0348 (7)	
H25	0.6523	0.6000	0.9684	0.042*	
C26	0.5680 (5)	0.7727 (3)	1.03684 (14)	0.0349 (7)	
H26	0.4508	0.7239	1.0430	0.042*	
C27	0.7411 (5)	0.7534 (3)	0.99881 (13)	0.0334 (7)	
C28	0.8673 (5)	0.8510 (3)	1.00599 (14)	0.0369 (7)	
H28	0.9992	0.8632	0.9851	0.044*	
C29	0.4592 (5)	0.9232 (3)	1.10852 (13)	0.0326 (7)	
C30	0.2761 (5)	0.8690 (3)	1.12765 (15)	0.0439 (8)	
H30	0.2409	0.7980	1.1116	0.053*	
C31	0.1439 (6)	0.9192 (4)	1.17057 (16)	0.0490 (9)	
H31	0.0161	0.8831	1.1833	0.059*	
C32	0.1950 (6)	1.0208 (3)	1.19508 (15)	0.0415 (8)	
H32	0.1048	1.0538	1.2249	0.050*	
C33	0.3778 (6)	1.0733 (3)	1.17562 (15)	0.0444 (8)	
H33	0.4132	1.1438	1.1920	0.053*	
C34	0.5120 (6)	1.0256 (3)	1.13250 (15)	0.0414 (8)	
H34	0.6388	1.0627	1.1195	0.050*	
C3	0.2541 (10)	1.1187 (6)	0.3523 (3)	0.0381 (17)	0.684 (10)
H3A	0.1785	1.1671	0.3131	0.046*	0.684 (10)
H3B	0.1613	1.1486	0.3846	0.046*	0.684 (10)
C4	0.2715 (9)	0.9670 (5)	0.3587 (3)	0.0426 (16)	0.684 (10)
H4A	0.3534	0.9378	0.3246	0.051*	0.684 (10)
H4B	0.1198	0.9474	0.3574	0.051*	0.684 (10)
C7	0.5375 (19)	1.2686 (11)	0.3194 (4)	0.135 (5)	0.684 (10)
H7A	0.6956	1.2674	0.3270	0.202*	0.684 (10)
H7B	0.4459	1.3503	0.3285	0.202*	0.684 (10)
H7C	0.5123	1.2677	0.2769	0.202*	0.684 (10)
C20	1.2497 (11)	0.5233 (8)	0.8404 (4)	0.054 (2)	0.636 (10)
H20A	1.3110	0.5201	0.8002	0.065*	0.636 (10)
H20B	1.3569	0.4605	0.8711	0.065*	0.636 (10)
C21	1.2252 (13)	0.6658 (7)	0.8506 (3)	0.054 (2)	0.636 (10)
H21A	1.1300	0.7296	0.8175	0.065*	0.636 (10)
H21B	1.3741	0.6900	0.8493	0.065*	0.636 (10)
C24	0.8901 (11)	0.4964 (9)	0.7912 (3)	0.055 (2)	0.636 (10)
H24A	0.7938	0.4300	0.7980	0.083*	0.636 (10)
H24B	0.9839	0.4844	0.7569	0.083*	0.636 (10)
H24C	0.7975	0.5875	0.7826	0.083*	0.636 (10)
C3'	0.304 (2)	1.0840 (16)	0.3361 (5)	0.042 (4)	0.316 (10)
H3'A	0.1804	1.1527	0.3134	0.050*	0.316 (10)
H3'B	0.3736	1.0270	0.3085	0.050*	0.316 (10)
C4'	0.2123 (16)	0.9985 (11)	0.3874 (6)	0.045 (4)*	0.316 (10)
H4'A	0.0937	0.9600	0.3723	0.054*	0.316 (10)
H4'B	0.1455	1.0540	0.4159	0.054*	0.316 (10)
C7'	0.6175 (13)	1.1961 (9)	0.3041 (3)	0.021 (2)*	0.316 (10)
H7'A	0.7578	1.2121	0.3189	0.031*	0.316 (10)
H7'B	0.5363	1.2789	0.2775	0.031*	0.316 (10)
H7'C	0.6501	1.1242	0.2816	0.031*	0.316 (10)
C20'	1.1615 (18)	0.5886 (11)	0.8212 (4)	0.039 (3)	0.364 (10)
H20C	1.2677	0.5612	0.7910	0.047*	0.364 (10)
H20D	1.0581	0.6731	0.8017	0.047*	0.364 (10)
C21'	1.2891 (14)	0.6119 (12)	0.8744 (5)	0.037 (3)	0.364 (10)
H21C	1.3702	0.5248	0.8989	0.044*	0.364 (10)
H21D	1.3989	0.6686	0.8596	0.044*	0.364 (10)
C24'	0.916 (3)	0.4338 (18)	0.7979 (5)	0.087 (6)	0.364 (10)
H24D	0.8396	0.3621	0.8171	0.130*	0.364 (10)
H24E	1.0261	0.3997	0.7705	0.130*	0.364 (10)
H24F	0.8067	0.5101	0.7750	0.130*	0.364 (10)

### Atomic displacement parameters (Å^2^)

**Table:**

	*U*^11^	*U*^22^	*U*^33^	*U*^12^	*U*^13^	*U*^23^
O1	0.0430 (14)	0.0421 (14)	0.0690 (17)	−0.0242 (11)	−0.0029 (12)	0.0011 (12)
O2	0.0393 (13)	0.0510 (14)	0.0572 (15)	−0.0256 (11)	0.0085 (11)	−0.0189 (12)
N1	0.0263 (13)	0.0344 (14)	0.0452 (16)	−0.0107 (11)	−0.0039 (12)	−0.0084 (12)
N2	0.0234 (12)	0.0321 (13)	0.0324 (14)	−0.0090 (10)	0.0019 (10)	−0.0084 (11)
N3	0.0327 (14)	0.0367 (15)	0.0458 (16)	−0.0152 (12)	0.0002 (12)	−0.0012 (13)
N4	0.0311 (14)	0.0322 (14)	0.0365 (15)	−0.0136 (11)	−0.0047 (11)	−0.0013 (11)
C1	0.0305 (17)	0.0417 (19)	0.047 (2)	−0.0142 (15)	0.0048 (15)	0.0015 (16)
C2	0.044 (2)	0.076 (3)	0.081 (3)	−0.031 (2)	−0.013 (2)	0.034 (2)
C5	0.0378 (18)	0.0356 (17)	0.046 (2)	−0.0123 (14)	−0.0051 (15)	−0.0097 (15)
C6	0.0293 (16)	0.0352 (17)	0.0364 (17)	−0.0115 (13)	0.0055 (14)	−0.0078 (14)
C8	0.0284 (16)	0.0372 (17)	0.0351 (17)	−0.0138 (13)	0.0071 (13)	−0.0105 (14)
C9	0.0228 (15)	0.0344 (16)	0.0384 (17)	−0.0111 (12)	0.0064 (13)	−0.0128 (14)
C10	0.0265 (15)	0.0330 (16)	0.0328 (16)	−0.0095 (13)	0.0048 (13)	−0.0109 (13)
C11	0.0299 (16)	0.0343 (17)	0.0367 (18)	−0.0098 (13)	−0.0032 (14)	−0.0082 (14)
C12	0.0251 (15)	0.0367 (17)	0.0297 (16)	−0.0021 (13)	0.0039 (13)	−0.0085 (13)
C13	0.0300 (17)	0.060 (2)	0.043 (2)	−0.0142 (16)	0.0006 (15)	−0.0015 (17)
C14	0.0299 (18)	0.076 (3)	0.044 (2)	−0.0071 (18)	−0.0025 (16)	−0.0081 (19)
C15	0.048 (2)	0.056 (2)	0.0375 (19)	0.0061 (18)	−0.0018 (17)	−0.0101 (17)
C16	0.063 (2)	0.0353 (19)	0.041 (2)	−0.0049 (17)	−0.0053 (18)	−0.0072 (15)
C17	0.0412 (18)	0.0371 (18)	0.0382 (18)	−0.0083 (14)	−0.0027 (15)	−0.0123 (15)
C18	0.0291 (17)	0.052 (2)	0.044 (2)	−0.0163 (15)	0.0012 (15)	−0.0128 (16)
C19	0.049 (2)	0.112 (4)	0.061 (3)	−0.045 (2)	0.019 (2)	−0.047 (2)
C22	0.0311 (17)	0.0359 (18)	0.058 (2)	−0.0143 (14)	0.0004 (16)	−0.0026 (16)
C23	0.0288 (16)	0.0362 (17)	0.0364 (18)	−0.0121 (13)	−0.0058 (14)	0.0012 (14)
C25	0.0316 (16)	0.0374 (17)	0.0359 (17)	−0.0163 (14)	−0.0037 (14)	−0.0005 (14)
C26	0.0327 (17)	0.0336 (17)	0.0389 (18)	−0.0160 (14)	−0.0045 (14)	−0.0004 (14)
C27	0.0311 (16)	0.0343 (17)	0.0337 (17)	−0.0123 (13)	−0.0056 (13)	0.0000 (14)
C28	0.0337 (17)	0.0390 (18)	0.0394 (18)	−0.0163 (14)	0.0000 (14)	−0.0034 (15)
C29	0.0333 (16)	0.0320 (16)	0.0299 (16)	−0.0072 (13)	−0.0045 (13)	−0.0002 (13)
C30	0.0383 (18)	0.052 (2)	0.049 (2)	−0.0198 (16)	0.0008 (16)	−0.0180 (17)
C31	0.0371 (19)	0.062 (2)	0.054 (2)	−0.0178 (17)	0.0030 (17)	−0.0201 (19)
C32	0.0423 (19)	0.0391 (18)	0.0384 (19)	−0.0016 (15)	−0.0043 (15)	−0.0047 (15)
C33	0.063 (2)	0.0317 (17)	0.0393 (19)	−0.0125 (16)	−0.0009 (17)	−0.0074 (15)
C34	0.049 (2)	0.0315 (17)	0.043 (2)	−0.0183 (15)	−0.0006 (16)	0.0008 (15)
C3	0.049 (4)	0.038 (3)	0.033 (4)	−0.014 (3)	−0.006 (3)	−0.012 (3)
C4	0.044 (3)	0.038 (3)	0.044 (3)	0.003 (2)	−0.011 (3)	−0.015 (2)
C7	0.197 (11)	0.124 (9)	0.086 (6)	−0.104 (9)	−0.040 (7)	0.036 (6)
C20	0.042 (4)	0.068 (5)	0.058 (5)	−0.030 (4)	0.006 (3)	−0.010 (4)
C21	0.034 (4)	0.049 (4)	0.069 (5)	−0.005 (3)	0.018 (4)	0.003 (4)
C24	0.051 (4)	0.065 (5)	0.042 (4)	0.010 (3)	0.002 (3)	−0.014 (3)
C3'	0.059 (7)	0.040 (7)	0.034 (7)	−0.012 (6)	−0.011 (5)	−0.024 (5)
C20'	0.041 (6)	0.038 (6)	0.038 (5)	−0.006 (5)	0.010 (4)	−0.008 (4)
C21'	0.025 (5)	0.030 (6)	0.055 (6)	−0.009 (4)	0.003 (4)	−0.006 (5)
C24'	0.112 (10)	0.088 (10)	0.091 (9)	−0.064 (8)	0.022 (7)	−0.047 (7)

### Geometric parameters (Å, °)

**Table:**

O1—C1	1.217 (4)	C22—C21	1.547 (6)
O2—C18	1.220 (4)	C22—H22A	0.9900
N1—C11	1.326 (4)	C22—H22B	0.9900
N1—N2	1.367 (3)	C23—C25	1.339 (4)
N2—C9	1.350 (4)	C25—C27	1.445 (4)
N2—C12	1.415 (4)	C25—H25	0.9500
N3—C28	1.322 (4)	C26—C27	1.374 (4)
N3—N4	1.373 (3)	C26—H26	0.9500
N4—C26	1.355 (4)	C27—C28	1.426 (4)
N4—C29	1.417 (4)	C28—H28	0.9500
C1—C6	1.485 (4)	C29—C30	1.378 (4)
C1—C2	1.497 (5)	C29—C34	1.386 (4)
C2—C7	1.395 (6)	C30—C31	1.385 (5)
C2—C7'	1.485 (7)	C30—H30	0.9500
C2—C3	1.504 (6)	C31—C32	1.381 (5)
C2—C3'	1.533 (8)	C31—H31	0.9500
C2—H1	1.0000	C32—C33	1.369 (5)
C2—H1'	1.0000	C32—H32	0.9500
C5—C4'	1.501 (8)	C33—C34	1.385 (5)
C5—C6	1.513 (4)	C33—H33	0.9500
C5—C4	1.559 (5)	C34—H34	0.9500
C5—H5A	0.9900	C3—C4	1.532 (6)
C5—H5B	0.9900	C3—H3A	0.9900
C5—H5C	0.9900	C3—H3B	0.9900
C5—H5D	0.9900	C4—H4A	0.9900
C6—C8	1.337 (4)	C4—H4B	0.9900
C8—C10	1.442 (4)	C7—H7A	0.9800
C8—H8	0.9500	C7—H7B	0.9800
C9—C10	1.381 (4)	C7—H7C	0.9800
C9—H9	0.9500	C20—C21	1.524 (8)
C10—C11	1.418 (4)	C20—H20A	0.9900
C11—H11	0.9500	C20—H20B	0.9900
C12—C17	1.380 (4)	C21—H21A	0.9900
C12—C13	1.390 (4)	C21—H21B	0.9900
C13—C14	1.378 (5)	C24—H24A	0.9800
C13—H13	0.9500	C24—H24B	0.9800
C14—C15	1.382 (5)	C24—H24C	0.9800
C14—H14	0.9500	C3'—C4'	1.486 (9)
C15—C16	1.384 (5)	C3'—H3'A	0.9900
C15—H15	0.9500	C3'—H3'B	0.9900
C16—C17	1.390 (4)	C4'—H4'A	0.9900
C16—H16	0.9500	C4'—H4'B	0.9900
C17—H17	0.9500	C7'—H7'A	0.9800
C18—C23	1.486 (4)	C7'—H7'B	0.9800
C18—C19	1.507 (5)	C7'—H7'C	0.9800
C19—C24	1.433 (7)	C20'—C21'	1.530 (9)
C19—C24'	1.462 (8)	C20'—H20C	0.9900
C19—C20	1.509 (6)	C20'—H20D	0.9900
C19—C20'	1.533 (8)	C21'—H21C	0.9900
C19—H19	1.0000	C21'—H21D	0.9900
C19—H19'	1.0000	C24'—H24D	0.9800
C22—C21'	1.497 (8)	C24'—H24E	0.9800
C22—C23	1.502 (4)	C24'—H24F	0.9800
			
C11—N1—N2	105.1 (2)	C26—C27—C25	122.4 (3)
C9—N2—N1	111.1 (2)	C28—C27—C25	134.1 (3)
C9—N2—C12	127.9 (2)	N3—C28—C27	112.2 (3)
N1—N2—C12	120.9 (2)	N3—C28—H28	123.9
C28—N3—N4	104.9 (2)	C27—C28—H28	123.9
C26—N4—N3	111.1 (3)	C30—C29—C34	120.4 (3)
C26—N4—C29	128.0 (2)	C30—C29—N4	119.4 (3)
N3—N4—C29	120.9 (2)	C34—C29—N4	120.3 (3)
O1—C1—C6	122.0 (3)	C29—C30—C31	119.3 (3)
O1—C1—C2	118.8 (3)	C29—C30—H30	120.4
C6—C1—C2	119.1 (3)	C31—C30—H30	120.4
C7—C2—C1	120.9 (4)	C32—C31—C30	121.1 (3)
C7'—C2—C1	111.0 (4)	C32—C31—H31	119.5
C1—C2—C3	114.4 (3)	C30—C31—H31	119.5
C7'—C2—C3'	109.2 (6)	C33—C32—C31	118.9 (3)
C1—C2—C3'	114.7 (6)	C33—C32—H32	120.6
C7'—C2—H1'	107.2	C31—C32—H32	120.6
C1—C2—H1'	107.2	C32—C33—C34	121.3 (3)
C3'—C2—H1'	107.2	C32—C33—H33	119.4
C4'—C5—C6	113.6 (5)	C34—C33—H33	119.4
C6—C5—C4	115.3 (3)	C29—C34—C33	119.2 (3)
C6—C5—H5A	108.4	C29—C34—H34	120.4
C4—C5—H5A	108.4	C33—C34—H34	120.4
C6—C5—H5B	108.4	C2—C3—C4	112.4 (5)
C4—C5—H5B	108.4	C2—C3—H3A	109.1
H5A—C5—H5B	107.5	C4—C3—H3A	109.1
C4'—C5—H5C	108.8	C2—C3—H3B	109.1
C6—C5—H5C	108.8	C4—C3—H3B	109.1
C4'—C5—H5D	108.8	H3A—C3—H3B	107.9
C6—C5—H5D	108.8	C3—C4—C5	110.6 (4)
H5C—C5—H5D	107.7	C3—C4—H4A	109.5
C8—C6—C1	116.7 (3)	C5—C4—H4A	109.5
C8—C6—C5	123.7 (3)	C3—C4—H4B	109.5
C1—C6—C5	119.6 (3)	C5—C4—H4B	109.5
C6—C8—C10	130.2 (3)	H4A—C4—H4B	108.1
C6—C8—H8	114.9	C2—C7—H7A	109.5
C10—C8—H8	114.9	C2—C7—H7B	109.5
N2—C9—C10	108.3 (2)	H7A—C7—H7B	109.5
N2—C9—H9	125.8	C2—C7—H7C	109.5
C10—C9—H9	125.8	H7A—C7—H7C	109.5
C9—C10—C11	103.4 (3)	H7B—C7—H7C	109.5
C9—C10—C8	122.5 (3)	C19—C20—C21	111.9 (6)
C11—C10—C8	134.0 (3)	C19—C20—H20A	109.2
N1—C11—C10	112.1 (3)	C21—C20—H20A	109.2
N1—C11—H11	124.0	C19—C20—H20B	109.2
C10—C11—H11	124.0	C21—C20—H20B	109.2
C17—C12—C13	120.1 (3)	H20A—C20—H20B	107.9
C17—C12—N2	119.7 (3)	C20—C21—C22	112.7 (5)
C13—C12—N2	120.2 (3)	C20—C21—H21A	109.1
C14—C13—C12	120.1 (3)	C22—C21—H21A	109.1
C14—C13—H13	119.9	C20—C21—H21B	109.1
C12—C13—H13	119.9	C22—C21—H21B	109.1
C13—C14—C15	120.5 (3)	H21A—C21—H21B	107.8
C13—C14—H14	119.8	C19—C24—H24A	109.5
C15—C14—H14	119.8	C19—C24—H24B	109.5
C14—C15—C16	119.1 (3)	H24A—C24—H24B	109.5
C14—C15—H15	120.5	C19—C24—H24C	109.5
C16—C15—H15	120.5	H24A—C24—H24C	109.5
C15—C16—C17	121.1 (3)	H24B—C24—H24C	109.5
C15—C16—H16	119.4	C4'—C3'—C2	113.2 (9)
C17—C16—H16	119.4	C4'—C3'—H3'A	108.9
C12—C17—C16	119.1 (3)	C2—C3'—H3'A	108.9
C12—C17—H17	120.5	C4'—C3'—H3'B	108.9
C16—C17—H17	120.5	C2—C3'—H3'B	108.9
O2—C18—C23	122.2 (3)	H3'A—C3'—H3'B	107.8
O2—C18—C19	118.5 (3)	C3'—C4'—C5	109.6 (10)
C23—C18—C19	119.2 (3)	C3'—C4'—H4'A	109.7
C24—C19—C20	121.1 (5)	C5—C4'—H4'A	109.7
C24—C19—C18	113.0 (4)	C3'—C4'—H4'B	109.7
C24'—C19—C18	116.8 (6)	C5—C4'—H4'B	109.7
C20—C19—C18	114.6 (4)	H4'A—C4'—H4'B	108.2
C24'—C19—C20'	116.0 (6)	C2—C7'—H7'A	109.5
C18—C19—C20'	112.2 (5)	C2—C7'—H7'B	109.5
C24—C19—H19	101.3	H7'A—C7'—H7'B	109.5
C20—C19—H19	101.3	C2—C7'—H7'C	109.5
C18—C19—H19	101.3	H7'A—C7'—H7'C	109.5
C24'—C19—H19'	103.1	H7'B—C7'—H7'C	109.5
C18—C19—H19'	103.1	C21'—C20'—C19	109.2 (8)
C20'—C19—H19'	103.1	C21'—C20'—H20C	109.8
C21'—C22—C23	113.7 (5)	C19—C20'—H20C	109.8
C23—C22—C21	114.2 (4)	C21'—C20'—H20D	109.8
C23—C22—H22A	108.7	C19—C20'—H20D	109.8
C21—C22—H22A	108.7	H20C—C20'—H20D	108.3
C23—C22—H22B	108.7	C22—C21'—C20'	107.5 (8)
C21—C22—H22B	108.7	C22—C21'—H21C	110.2
H22A—C22—H22B	107.6	C20'—C21'—H21C	110.2
C25—C23—C18	115.9 (3)	C22—C21'—H21D	110.2
C25—C23—C22	124.0 (3)	C20'—C21'—H21D	110.2
C18—C23—C22	120.1 (3)	H21C—C21'—H21D	108.5
C23—C25—C27	131.0 (3)	C19—C24'—H24D	109.5
C23—C25—H25	114.5	C19—C24'—H24E	109.5
C27—C25—H25	114.5	H24D—C24'—H24E	109.5
N4—C26—C27	108.4 (3)	C19—C24'—H24F	109.5
N4—C26—H26	125.8	H24D—C24'—H24F	109.5
C27—C26—H26	125.8	H24E—C24'—H24F	109.5
C26—C27—C28	103.5 (3)		
			
C11—N1—N2—C9	0.2 (3)	C21'—C22—C23—C25	172.6 (5)
C11—N1—N2—C12	176.0 (2)	C21—C22—C23—C25	−156.8 (4)
C28—N3—N4—C26	0.0 (3)	C21'—C22—C23—C18	−6.3 (6)
C28—N3—N4—C29	177.7 (3)	C21—C22—C23—C18	24.3 (5)
O1—C1—C2—C7	19.2 (10)	C18—C23—C25—C27	178.7 (3)
C6—C1—C2—C7	−160.0 (8)	C22—C23—C25—C27	−0.1 (5)
O1—C1—C2—C7'	60.4 (6)	N3—N4—C26—C27	0.2 (3)
C6—C1—C2—C7'	−118.8 (5)	C29—N4—C26—C27	−177.2 (3)
O1—C1—C2—C3	−149.8 (4)	N4—C26—C27—C28	−0.3 (3)
C6—C1—C2—C3	31.0 (6)	N4—C26—C27—C25	178.5 (3)
O1—C1—C2—C3'	−175.3 (7)	C23—C25—C27—C26	−178.2 (3)
C6—C1—C2—C3'	5.6 (8)	C23—C25—C27—C28	0.1 (6)
O1—C1—C6—C8	−14.5 (5)	N4—N3—C28—C27	−0.2 (3)
C2—C1—C6—C8	164.6 (3)	C26—C27—C28—N3	0.3 (3)
O1—C1—C6—C5	163.7 (3)	C25—C27—C28—N3	−178.2 (3)
C2—C1—C6—C5	−17.2 (5)	C26—N4—C29—C30	−1.2 (4)
C4'—C5—C6—C8	166.4 (6)	N3—N4—C29—C30	−178.4 (3)
C4—C5—C6—C8	−158.6 (4)	C26—N4—C29—C34	178.7 (3)
C4'—C5—C6—C1	−11.6 (7)	N3—N4—C29—C34	1.6 (4)
C4—C5—C6—C1	23.4 (5)	C34—C29—C30—C31	0.8 (5)
C1—C6—C8—C10	177.7 (3)	N4—C29—C30—C31	−179.2 (3)
C5—C6—C8—C10	−0.4 (5)	C29—C30—C31—C32	−1.1 (5)
N1—N2—C9—C10	0.0 (3)	C30—C31—C32—C33	0.9 (5)
C12—N2—C9—C10	−175.3 (3)	C31—C32—C33—C34	−0.5 (5)
N2—C9—C10—C11	−0.3 (3)	C30—C29—C34—C33	−0.4 (5)
N2—C9—C10—C8	177.2 (3)	N4—C29—C34—C33	179.6 (3)
C6—C8—C10—C9	−178.4 (3)	C32—C33—C34—C29	0.3 (5)
C6—C8—C10—C11	−1.8 (6)	C7—C2—C3—C4	139.6 (8)
N2—N1—C11—C10	−0.4 (3)	C7'—C2—C3—C4	92.8 (7)
C9—C10—C11—N1	0.5 (3)	C1—C2—C3—C4	−51.8 (8)
C8—C10—C11—N1	−176.6 (3)	C3'—C2—C3—C4	44.3 (15)
C9—N2—C12—C17	174.6 (3)	C2—C3—C4—C5	57.3 (8)
N1—N2—C12—C17	−0.4 (4)	C4'—C5—C4—C3	51.7 (8)
C9—N2—C12—C13	−4.0 (4)	C6—C5—C4—C3	−42.7 (6)
N1—N2—C12—C13	−179.0 (3)	C24—C19—C20—C21	92.5 (9)
C17—C12—C13—C14	−1.5 (5)	C24'—C19—C20—C21	121.6 (13)
N2—C12—C13—C14	177.1 (3)	C18—C19—C20—C21	−48.6 (9)
C12—C13—C14—C15	0.4 (5)	C20'—C19—C20—C21	43.9 (8)
C13—C14—C15—C16	1.0 (5)	C19—C20—C21—C22	56.7 (10)
C14—C15—C16—C17	−1.4 (5)	C21'—C22—C21—C20	51.3 (9)
C13—C12—C17—C16	1.2 (5)	C23—C22—C21—C20	−44.0 (8)
N2—C12—C17—C16	−177.5 (3)	C7—C2—C3'—C4'	−160.3 (10)
C15—C16—C17—C12	0.3 (5)	C7'—C2—C3'—C4'	159.8 (10)
O2—C18—C19—C24	63.2 (6)	C1—C2—C3'—C4'	34.5 (15)
C23—C18—C19—C24	−115.3 (5)	C3—C2—C3'—C4'	−60.1 (12)
O2—C18—C19—C24'	36.2 (10)	C2—C3'—C4'—C5	−63.2 (17)
C23—C18—C19—C24'	−142.4 (9)	C6—C5—C4'—C3'	50.6 (13)
O2—C18—C19—C20	−152.5 (5)	C4—C5—C4'—C3'	−49.8 (8)
C23—C18—C19—C20	29.0 (6)	C24—C19—C20'—C21'	167.5 (9)
O2—C18—C19—C20'	173.6 (5)	C24'—C19—C20'—C21'	−174.0 (12)
C23—C18—C19—C20'	−5.0 (7)	C20—C19—C20'—C21'	−53.1 (8)
O2—C18—C23—C25	−14.5 (5)	C18—C19—C20'—C21'	48.2 (10)
C19—C18—C23—C25	164.0 (3)	C23—C22—C21'—C20'	48.9 (10)
O2—C18—C23—C22	164.5 (3)	C21—C22—C21'—C20'	−48.4 (8)
C19—C18—C23—C22	−17.1 (5)	C19—C20'—C21'—C22	−71.6 (12)

### Hydrogen-bond geometry (Å, °)

**Table:**

*D*—H···*A*	*D*—H	H···*A*	*D*···*A*	*D*—H···*A*
C9—H9···O1^i^	0.95	2.29	3.157 (4)	152
C26—H26···O2^ii^	0.95	2.30	3.224 (4)	164
C30—H30···O2^ii^	0.95	2.57	3.506 (4)	167

Symmetry codes: (i) −*x*+2, −*y*+2, −*z*+1; (ii) −*x*+1, −*y*+1, −*z*+2.

## References

[bb1] Asiri, A. M. & Khan, S. A. (2010). *Molbank*, **M681**, 3.

[bb2] Barbour, L. J. (2001). *J. Supramol. Chem.* **1**, 189–191.

[bb3] Bruker (2009). *APEX2* and *SAINT* Bruker AXS Inc., Madison, Wisconsin, USA.

[bb4] Sheldrick, G. M. (2008). *Acta Cryst.* A**64**, 112–122. 10.1107/S0108767307043930 18156677

[bb5] Westrip, S. P. (2010). *J. Appl. Cryst.* **43**, 920–925.

